# Investor sentiment-aware prediction model for P2P lending indicators based on LSTM

**DOI:** 10.1371/journal.pone.0262539

**Published:** 2022-01-27

**Authors:** Yanyan Cui, Lixin Liu

**Affiliations:** School of Statistics, University of International Business and Economics, Beijing, China; Shandong University of Science and Technology, CHINA

## Abstract

In recent years, online lending has created many risks while providing lending convenience to Chinese individuals and small and medium-sized enterprises. The timely assessment and prediction of the status of industry indicators is an important prerequisite for effectively preventing the spread of risks in China’s new financial formats. The role of investor sentiment should not be underestimated. We first use the BERT model to divide investor sentiment in the review information of China’s online lending third-party information website into three categories and analyze the relationship between investor sentiment and quantitative indicators of online lending product transactions. The results show that the percentage of positive comments has a positive relationship to the borrowing interest rate of P2P platforms that investors are willing to participate in for bidding projects. The percentage of negative comments has an inverse relationship to the borrowing period. Second, after introducing investor sentiment into the long short-term memory (LSTM) model, the average RMSE of the three forecast periods for borrowing interest rates is 0.373, and that of the borrowing period is 0.262, which are better than the values of other control models. Corresponding suggestions for the risk prevention of China’s new financial formats are made.

## 1 Introduction

P2P lending originated in the United Kingdom and is a lending method that relies on the internet environment to realize direct lending transactions between individuals and other individuals without financial institutions’ participation [1, 2]. China’s P2P lending environment has developed rapidly. From the emergence of the first P2P platform, Paipaidai, in 2007 to the end of 2019, the historical cumulative transaction volume of China’s P2P industry reached approximately 9 trillion yuan over more than ten years. In the U.S., the P2P industry transaction volume surpassed $5 billion over four years. This process took only two years in China. The vast Chinese financial market provides fertile ground for the development of fintech. The P2P lending method supported by fintech has also met the financial service needs of many SMEs and individuals in China to a certain extent. However, due to weak industry entry barriers and unsound platform risk supervision, the development of Chinese P2P lending has exposed many new financial format problems [3].

The Chinese P2P lending system shows that both lenders’ and borrowers’ product transaction data can directly reflect the industry’s current development, reveal possible hidden risks, and provide investors with good forecasting data. The average borrowing interest rate of P2P market products and the average borrowing period of transactions are two significant indicators [4]. The average borrowing interest rate of a product has been discussed by many scholars and is the core indicator of a P2P market product. It is generally believed that the higher the borrowers’ default risk level is, the higher the average borrowing interest rate of a product is, and the riskier P2P platforms are [5]. As an essential indicator of the transaction process, a transaction’s average borrowing period reflects the loan transaction period. Using this indicator also reflects the health of P2P platform transactions The above two indicators reflect the risks of a P2P platform to a certain extent. Suppose we can better predict the above two product transaction indicators. In this case, we can grasp P2P lending conditions and anticipate the risks of the P2P market more clearly. We can then sufficiently determine the future transformation and development of existing P2P platforms and provide better guidance strategies for P2P lending to serve SMEs and individuals’ loan needs.

In addition, due to the large number of P2P platforms in China, many P2P lending participants discuss their views on different P2P platforms and on the development of the P2P industry on P2P third-party information websites. These comments can express current investor sentiment and leave a mark on the internet. Whether it is possible to predict the indicators of the P2P market over time is a question worthy of attention. Therefore, we take the Chinese P2P industry as our research context, take the indicators of P2P market transactions as our research object, and propose the following two hypotheses:

1. There is a statistically causal relationship between P2P lending participants’ investor sentiment and the indicators of the P2P market.
2. Based on P2P lending participants’ investor sentiment, we can construct a model to predict the indicators of the P2P market.

**Fig 1:**
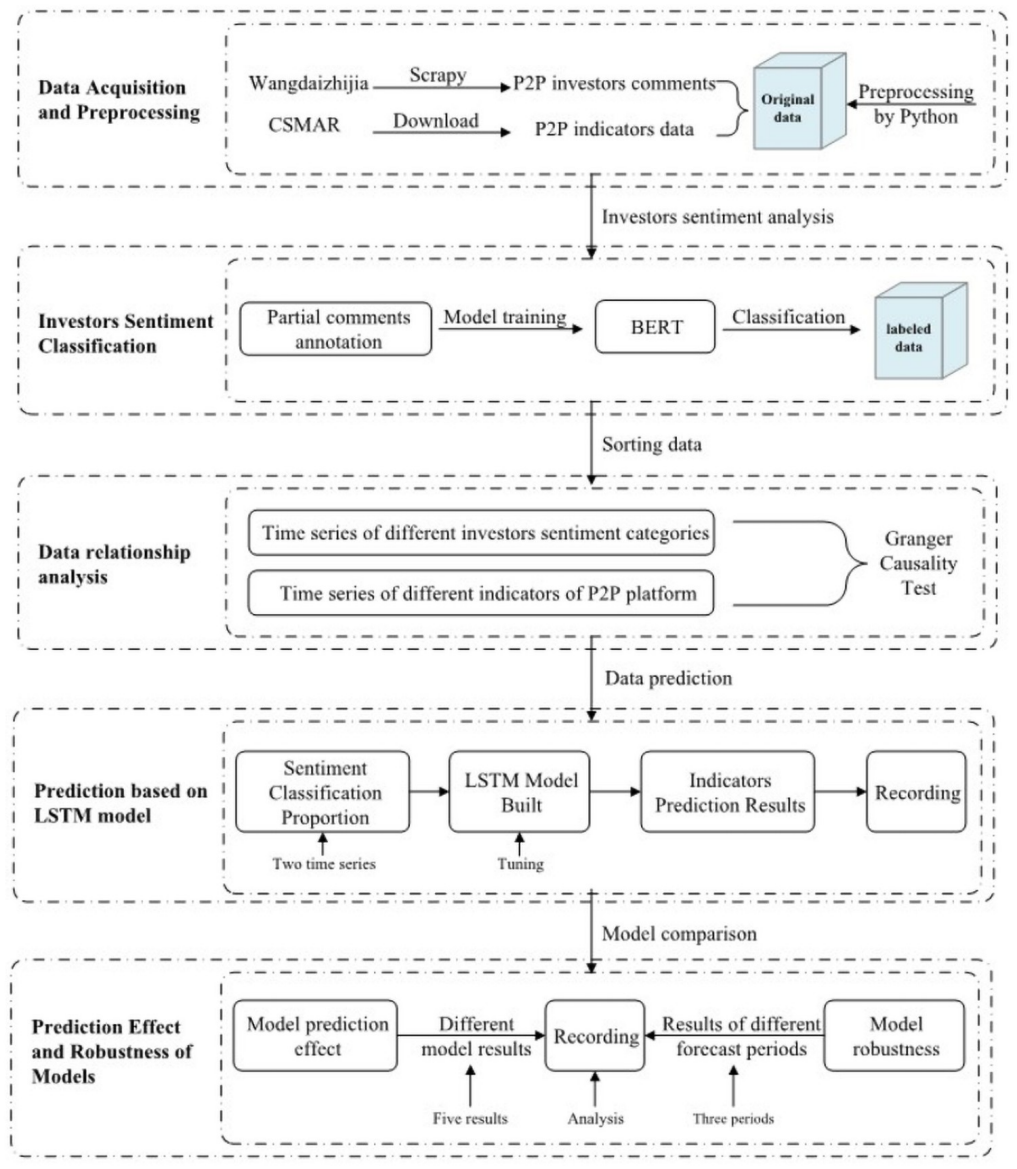
Research framework.

To test the above hypotheses, we first use Python software to crawl comments published by P2P third-party information website investors. In the constructed sentiment classification model, investors’ emotional expression is classified and sorted chronologically. Then, the categorized expression percentage of investor sentiment and the time series of the average borrowing interest rate and average borrowing period are tested by Granger causality. Finally, to predict the average borrowing interest rate and average borrowing period indicators of the P2P market, we use the LSTM model and compare it to other machine learning models. The details of this approach are shown in Fig 1.

The present article’s characteristics and innovations are as follows. First, unlike traditional forecasting methods, we directly use investor sentiment, which changes over time, to make predictions of P2P market indicator changes with more accurate results. Second, we use the Chinese P2P lending industry as a framework to relate the investor sentiment of P2P lending participants to P2P market indicators and construct a forecasting model, which addresses the gap in the research on investor sentiment in Chinese P2P lending indicator forecasting analysis. Finally, we help elucidate the development of China’s P2P industry and provide effective data evidence for anticipating the future development direction of China’s new financial formats and for formulating market strategies with the aid of financial technology.

The article’s structure is organized as follows. The second part provides a literature review of related papers. The third part outlines the acquisition, sorting and preprocessing of our sample data. The Granger causality test of the sentiment classification percentage and the indicator time series is also explained in this section. The fourth part presents our empirical analysis of the LSTM model and other comparison models in predicting the indicators of P2P platforms. Finally, conclusions are given in the fifth part, and relevant suggestions are put forward. Codes discussed in the empirical section of the article were based on GPU computing.

## 2 Literature review

### 2.1 On the problems of P2P lending

In the existing literature, there is a wealth of research on P2P lending, including discussion of the factors that affect P2P platform lending transactions, research on the risks of P2P platform borrowers, and exploration of the dangers of P2P platforms.

The development of fintech has prompted P2P platforms to help many SMEs and individuals conduct lending business. However, due to information asymmetry between borrowers and lenders, many factors affect lending transactions. Among these factors, the borrower’s social relationships are an essential signal of credit quality [6, 7]. Social relationships can increase the probability of successful borrowing and lower the interest rate of loans. Additionally, lenders tend to prefer borrowers who are geographically and culturally similar [8]. The content of borrowers’ descriptive information in terms of factors such as spelling errors, text length, and positive emotional keywords can also affect whether a lender borrows [9]. Related research has found beauty premiums in P2P lending behaviour, and a lender is more tolerant of the dishonest actions of attractive borrowers [10]. A lender will also show goodwill to a borrower who borrows to support public and charity-related undertakings and actively lower the loan interest rate [11].

In addition, various risks of borrowers on P2P platforms have also been widely discussed. Studies have shown that personal social relationships can reduce borrowers’ moral hazard to a certain extent [12]. Nevertheless, the structural social capital of borrowers hurts borrowing and repayment results [13]. Additionally, factors such as the borrower’s credit rating, debt-to-income ratio, FICO score, and borrowing cycle play an important role in evaluating loan default risks and require special attention [14, 15].

Finally, in terms of the risks of the P2P platform itself, empirical results have shown that the shareholder backgrounds, platform risk management level and institutional environment of the P2P platform are three crucial factors that affect the operational status of P2P platforms in China [16]. In particular, to enhance their competitiveness, some P2P platforms allow higher-risk borrowers to participate in borrowing activities, thereby increasing their risks [1].

In addition to exploring the above three aspects, some scholars have conducted investigations and studies of how lenders decide on lending behaviour based on limited information. The results show that when information about borrowers’ creditworthiness is extremely limited, lenders are more inclined to make a herd decision. When more relevant signals are transmitted through the market, lenders will follow their own judgement [17, 18].

### 2.2 Natural Language Processing (NLP) and deep learning technology

The extraction and classification of investor sentiment from comments is used in sentiment analysis in the field of NLP, which involves text and news classification, topic analysis, the use of question answering systems and natural language inference tasks [19]. With NLP development, models for training machines to understand human language have become increasingly abundant, and deep learning technology has played a significant role in promoting this development.

Generally, the development of NLP can be roughly divided into three stages. The first stage mainly includes the realization of word embedding [20] and representing human words through data quantification. Word2vec [21] and Global Vectors for Word Representation (GloVe) [22] are introducing word embedding into mainstream NLP.

The second stage involves more exploration of word sequence information extraction based on CNN and RNN approaches. CNN-based models use more convolutional structures to train models from space, whereas RNN-based models focus on memory over time [23]. Among the many model variants based on RNNs, the most widely used model is based on long short-term memory (LSTM) [24, 25]. Compared to the traditional RNN model, this model alleviates the gradient disappearance and gradient explosion problems of RNNs, can perform better over longer sequences and has been better applied and researched. Furthermore, the Embeddings from Language Model (ELMo) [26], which uses LSTM as a framework for feature extraction and is trained based on the language model, learns the correlations between word contexts well. The ability to dynamically discover the meanings of word vectors in different contexts has progressed NLP development a step further.

Finally, the third stage applies the attention mechanism. The transformer model uses the attention mechanism to replace RNN and performs well on multiple tasks [27]. Using the Transformer as the framework for feature extraction, the BERT model [28], which implements the Masked Language Model (MLM) and Next Sentence Prediction (NSP) in the pretraining step, has attracted widespread attention. This model learns the semantic information in a word sequence by masking some words in the corpus, refreshing the list of all NLP tasks for a time. Then, the improved RoBERTa [29] model and ALBERT [30] model, which take BERT as the prototype, both try to adjust the structure of different parameters to better apply the BERT model and achieve better performance in some aspects of NLP.

The LSTM model provides a foundation for NLP development and offers many avenues for model improvement in the financial field. Existing studies show that the results of an ensemble model that combines multiple LSTM models are better than the results of lasso and ridge regression in predicting daily stock prices [31]. The LSTM-CNN model, which extracts time and image features from stock time series and stock images to predict stock prices, can effectively reduce prediction error compared to a single method [32]. The hybrid forecasting model that combines EMD and LSTM models [33] also shows better performance in predicting major global stock indices’ daily closing prices.

### 2.3 Related research on investor sentiment

There are relatively few studies on the P2P industry’s investor sentiment in the existing literature, but the role of investor sentiment in economic problems cannot be underestimated. The empirical results show that investor sentiment on Twitter microblogs and the number of microblogs published are relevant to the S&P 500 index, to low market capitalization portfolios, and to earnings forecasts in specific industries [34]. The local attention and investor sentiment indicators extracted from East Money Internet Stock Bar comments significantly impact stock yields [35]. Negative media tone indicators representing Chinese IPO companies’ investors sentiment extracted from mainstream media reports can provide a reasonable explanation for the IPO underpricing rate, first-day turnover rate, and overraising ratio [36]. Companies that intend to go public can increase the optimism of investors by enhancing their positive advertising efforts, thereby increasing the price of securities issuance [37]. Investors’ online search behaviour can affect asset pricing in the stock market [38]. By constructing a corresponding sentiment index, this can effectively improve the forecasting effect of stock returns [39].

The above literature on investor sentiment mostly focuses on predicting quantitative indicators of the stock market, which are inseparable from factors such as the maturity of stock forum information and investment stocks’ followers. With the development of Chinese P2P lending, the industry has the same research conditions as the stock market. Wang and Huang use the constructed financial technology sentiment index to describe the media’s attention to the financial technology industry and then analyze the influence of media sentiment on the activities of the Chinese online lending market [40]. Fu *et al*. reveal the relationship between time series of sentiment changes in comments of Chinese P2P platform third-party information website Wangdaizhijia and the P2P platform transaction volume index. The authors in turn propose a model for forecasting the P2P platform trading volume index based on investor sentiment trends [41]. However, generally speaking, research on investor sentiment in the P2P industry is still limited.

In summary, we find that the existing literature on China’s P2P market still shows the following gaps. First, the overall transaction indicators of China’s P2P market are not sufficiently understood, and there is still a lack of relevant forecasting research. Second, research on investor sentiment in China’s P2P market is relatively limited, and the introduction of investor sentiment into the prediction model requires more consideration. To bridge these gaps, we attempt to use text classification models of NLP to analyze investor sentiment exhibited in internet comments and use this approach to predict the average borrowing interest rate and average borrowing period of the Chinese P2P market. Our study further explains the role of investor sentiment in China’s P2P industry and better explains the overall state of China’s P2P market.

## 3 Sample data processing and analysis

### 3.1 Data sources

There are many P2P third-party information websites in China, including Wangdaizhijia, Wangdaitianyan, and Diyiwangdai. Among them, the Wangdaizhijia website is China’s first online lending industry portal website. Since its development in 2011, it has gradually established China’s largest and most authoritative and influential P2P industry portals. Wangdaizhijia is committed to serving P2P investors, has set up a separate module for China’s P2P platform data and updated it regularly, had motivated many P2P investors to discuss and share opinions on its website, and has become a network gathering place for P2P investors. Therefore, we use comments left on Wangdaizhijia’s P2P platforms as a source of unstructured data to comprehensively reflect investors’ emotional state in China’s P2P industry. We use web crawler technology to obtain 154,205 comments written in Chinese and posted on 692 P2P platforms over 1182 days from September 27, 2015, to February 26, 2019.

The structured data of the quantitative indicators of this article come from the China Stock Market and Accounting Research (CSMAR) database. These structured data have not been further processed and more fully reflect each P2P platform’s conditions in different periods. Therefore, we matched the names of 692 Wangdaizhijia P2P platforms and collected data on the average borrowing interest rate and average borrowing period of these platforms from the CSMAR database.

### 3.2 Sentiment classification of comments

#### 1) Comments tagging

After considering the sample comments, we divide comment sentiment into negative, neutral and positive comments. Among these, negative comments refer to comments that cite dissatisfaction with the P2P platforms, disappointment with the P2P industry, doubts about the P2P platform executive team, and criticism of the P2P platforms’ business and services. Positive comments praise the development of P2P platforms and the P2P industry. Other comments are classified as neutral comments.

To apply the sentiment classification model, we randomly select 15,000 comments for labelling. The number -1 refers to negative statements in the dataset, the number 0 refers to neutral comments, and the number 1 refers to positive comments. The manual classification results show that among the 15,000 comments, there are 4387 negative comments, 2698 neutral comments, and 7915 positive comments.

#### 2) Sentiment classification model selection

After the comments are labelled, we employ a template used for computers to learn how humans perform classification. Then, the text classification model of NLP completes the process of teaching the computer. We use the BERT model as our sentiment classification model. The model’s training set represents 90% of the 15,000 comments, including content and label results, and the verification set represents the remaining 10%.

**Fig 2:**
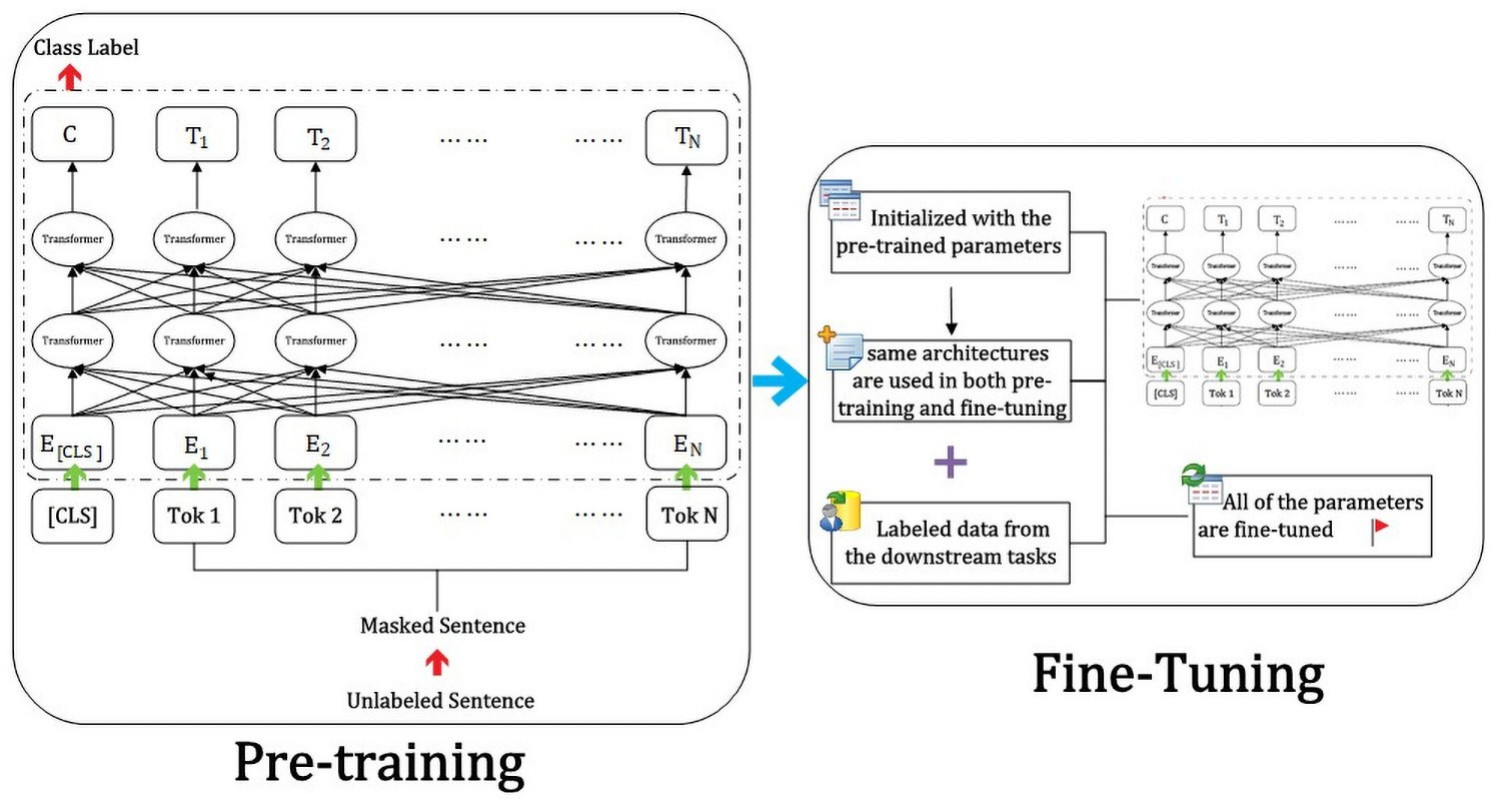
BERT process structure.

**Fig 3:**
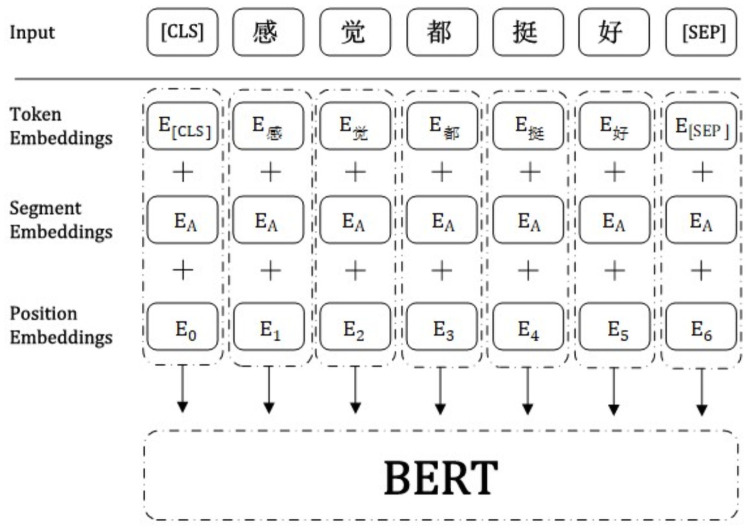
Schematic diagram of the BERT model input structure.

First, combined with the empirical data scenario, as the BERT model pretraining model, we use as the BERT-Base Chinese model, and the fine-tuning of downstream tasks is performed based on this model. The process is illustrated in Fig 2. Second, before training samples, the model first encodes the text input, including token embeddings, segment embeddings, and position embeddings, as shown in Fig 3.

**Fig 4:**
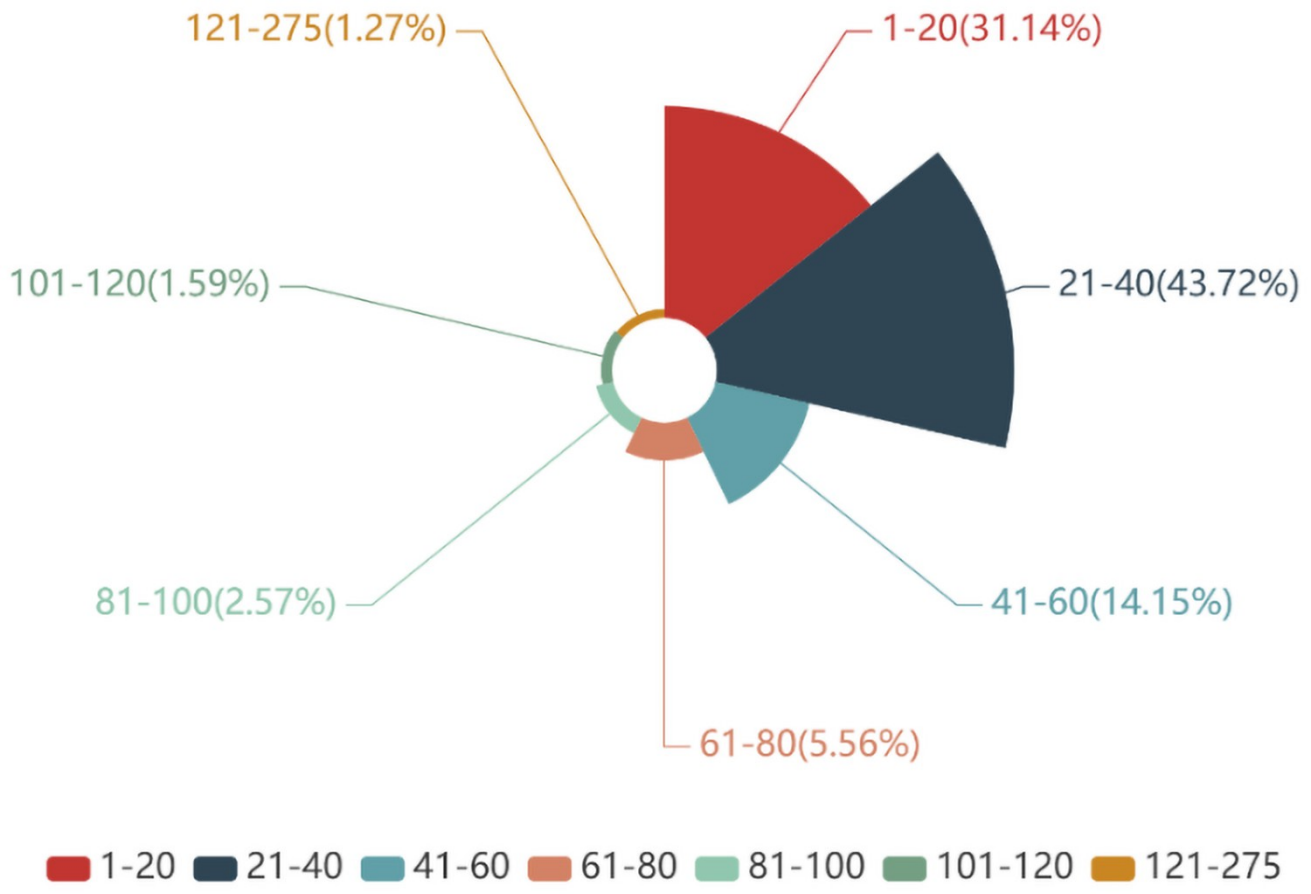
Comments text length.

Finally, we set the relevant parameters for model training. Most of the comments collected are 21–40 words long, and the longest comment is 275 words long (as shown in Fig 4), so we set the max_seq_length parameter of the BERT model to 275. After many trials and rounds of debugging, we set the train_bach_size parameter to 16, the learning_rate to 2e-5, and the num_train_epochs parameter to 4. The specific parameter structure employed and related descriptions are shown in Table 1.

**Table 1:** BERT model training specific parameters.

Parameter	Description	Value
max_seq_length	The maximum length of comments	275
train_batch_size	batch size	16
learning_rate	Learning rate	2e-5
num_train_epochs	training epochs	4

To evaluate the effect of the BERT model, we use accuracy, precision, recall and F1-score measures for characterization and use the TextCNN model for comparisons. The results show that the accuracy of the BERT model is 0.8579, the precision level is 0.8158, the recall level is 0.8579, and the F1-score is 0.8198. The accuracy of the TextCNN model is valued at 0.8027, the precision level is 0.7957, the recall level is 0.8027, and the F1-score is 0.7987. The above results demonstrate the advantages of the BERT model, so we use this model to classify the sentiment of all text comments collected. Finally, 43,874 negative comments, 27918 neutral comments, and 82413 positive comments are obtained.

#### 3) Granger causality test of investor sentiment and quantitative indicators

From simple calculations, we obtain the time series of the percentage of negative and positive comments left on 692 sample P2P platforms during the data collection period. To further explore the relationship between the time series of positive comments on investor sentiment and the time series of the average borrowing interest rate of the sampled platforms, we first standardize the data. After standardization, the Pearson correlation coefficient between the above two time series is 0.237, and the p value of the correlation is less than 0.1. Therefore, we propose Hypothesis 1.1 based on our first assumption and the Pearson correlation analysis results.

Hypothesis 1.1: There may be a positive relationship between the time series of the average borrowing interest rate and the time series of positive comments on investor sentiment. As the proportion of positive investor sentiment comments increases, the borrowing interest rate of investors willing to participate in bidding projects on P2P platforms will increase.

Similarly, after standardizing the percentage of negative investor sentiment comments and the average borrowing period time series of the sample platforms, the Pearson correlation coefficient is -0.258, and the p value of the correlation is less than 0.1. Therefore, we continue to propose Hypothesis 1.2 based on our first assumption.

Hypothesis 1.2: There may be an inverse relationship between the time series of the average borrowing period and the time series of negative comments on investor sentiment. As the proportion of negative investor sentiment comments increases, the borrowing period for investors willing to participate in bidding projects on P2P platforms will become shorter.

To demonstrate these hypotheses with more scientific rigor, we conduct a Granger causality test. As the prerequisite of the Granger causality test is a stationary test object time series, we first perform a unit root test of stationarity on the experimental time series. The null hypothesis reports a unit root or that the time series is not stationary. The calculation results are shown in Table 2.

**Table 2:** Unit root test results.

Time series	T value	P-value
Percentage of positive-sentiment	-9.3375	0.01
Average borrowing interest rate	-19.223	0.01
Percentage of negative-sentiment	-14.837	0.01
Average borrowing period	-8.268	0.01

As shown in Table 2, the values of the unit root test statistics of the four time series are less than the critical value of -3.96, and the P value results are less than 0.05. Thus, none of the four time series have unit roots, which means that the four time series used in the test are stable. Therefore, we can carry out the Granger causality test.

Hypothesis 1.1 Granger causality test null hypothesis: the time series of positive comments on investor sentiment is not the Granger cause of the time series of the average borrowing interest rate.

Hypothesis 1.2 Granger causality test null hypothesis: the time series of negative comments on investor sentiment is not the Granger cause of the time series of the average borrowing period.

The specific Granger causality test P value results are shown in Table 3.

**Table 3:** Granger causality test results.

Lagged value	1	2	3	4	5
p-value of hypothesis 1.1	0.0024	0.0109	0.0288	0.0324	0.0224
p-value of hypothesis 1.2	4.123e-07	0.0032	0.0054	0.0123	0.0054

Table 3 shows that the p value results of the Granger causality test are less than 0.05, so null Hypothesis 1.1 and null Hypothesis 1.2 are rejected. This result shows that the time series of positive comments on investor sentiment is the Granger cause of the time series of the average borrowing interest rate, which proves that the percentage of positive comments on investor sentiment can be used to predict the average borrowing interest rate of P2P platforms. This result also shows that the time series of negative comments on investor sentiment is the Granger cause of the time series of the average borrowing period, which proves that the percentage of negative comments on investor sentiment can be used to predict the average borrowing period of P2P platforms. Thus far, Hypothesis 1 is confirmed; that is, the investor sentiment of P2P lending participants has a statistically causal relationship to the quantitative indicators of the P2P market.

## 4 Empirical analysis

### 4.1 Prediction model selection and empirical results

**Fig 5:**
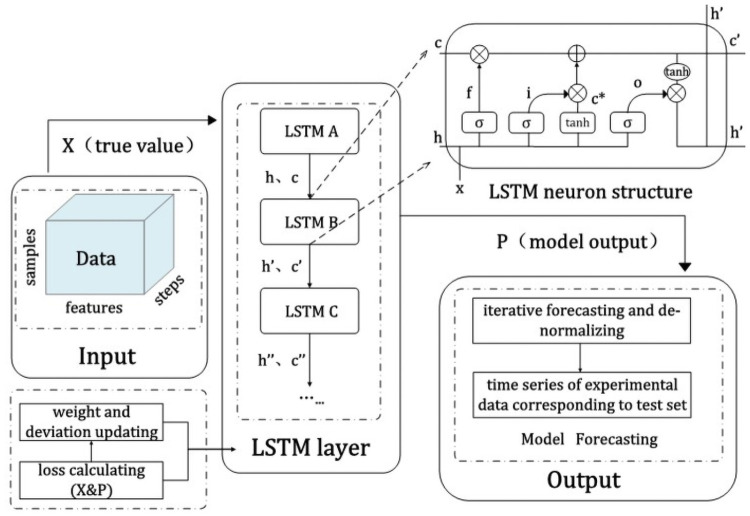
LSTM model training process.

To better use the time series of investor sentiment classification to predict the quantitative indicators of the P2P market, we use the LSTM model suitable for time series data forecasting as our preferred model. The model has a particular RNN structure and can perform well in terms of long-term memory. We illustrate the model training process in Fig 5. We (1) match the dataset’s LSTM input structure; (2) use forward propagation to calculate the output value of each LSTM neuron; (3) apply back propagation to calculate the error value of each LSTM neuron; (4) optimize and update different weight values according to the corresponding error values to obtain different parameter structures; (5) evaluate models with different parameter structures, and repeat steps (2)-(5) until the optimized LSTM structure is obtained.

We finally obtain 794 time series data from 692 sample P2P platforms for the data collection period after matching and preprocessing platform data. The first 700 pieces of data of this sample are taken as the training set, and the last 94 pieces of data are taken as the test set. In the model training process, we use the dropout method to prevent the LSTM model from overfitting. The specific model parameter settings are shown in Table 4.

**Table 4:** LSTM model training specific parameters.

Parameter	Description	Value
cell size	Number of hidden layer neurons	50
loss function	Choice of the loss function	Mean Absolute Error
optimizer	Determine the optimizer	Adam
batch size	The size of each batch of samples	20
learning rate	Learning rate value	0.001
N epoch	training epochs	1000

**Fig 6:**
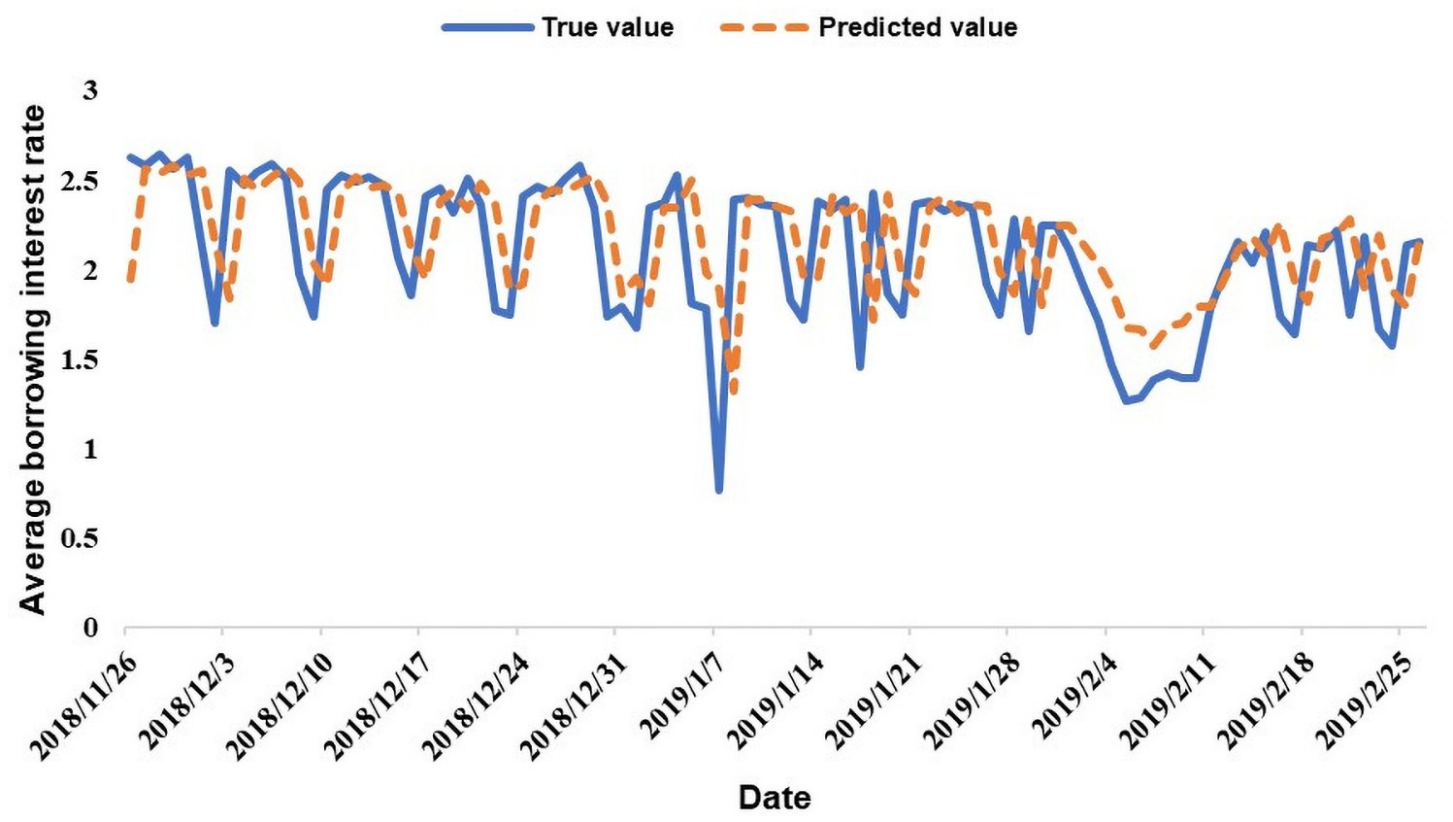
Average borrowing interest rate prediction results.

**Fig 7:**
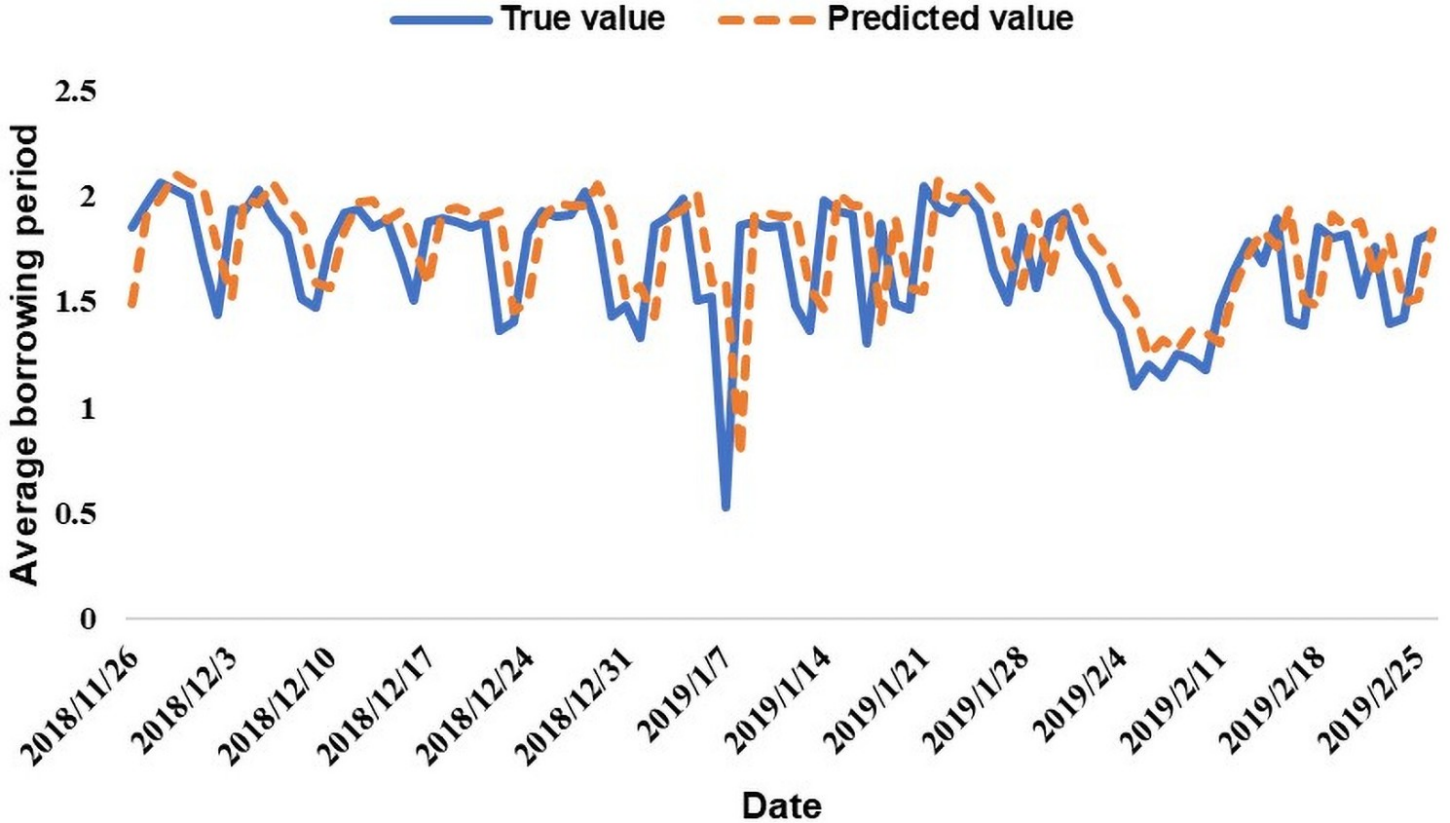
Average borrowing period prediction results.

The line graphs comparing the predicted value and the true value with 94 data points as the test set are shown in Figs 6 and 7. The dotted line represents the predicted value, and the solid line represents the true value.

Figs 6 and 7 show that the LSTM model better predicts the P2P market quantitative indicators for the 94 periods. Therefore, Hypothesis 2, which is based on P2P lending participants’ investor sentiment, proves that a model can be constructed to predict the quantitative indicators of the P2P market. However, to evaluate the prediction effect of the test set more specifically, we apply root mean square error (RMSE), mean absolute percentage error (MAPE) and symmetric mean absolute percentage error (SMAPE) tests to evaluate the model’s test set prediction effect. The specific formulas of the three evaluation indicators are as follows:

$$RMSE=\sqrt{\frac{1}{n}\sum_{i=1}^{n}{\left(\hat{y_{i}}-y_{i}\right)}^{2}}$$ (1)

$$MAPE=\frac{100\%}{n}\sum_{i=1}^{n}\left|\frac{\hat{y_{i}}-y_{i}}{y_{i}}\right|$$ (2)

$$SMAPE=\frac{100\%}{n}\sum_{i=1}^{n}\frac{\left|\hat{y_{i}}-y_{i}\right|}{\left(\left|\hat{y_{i}}\right|+\left|y_{i}\right|\right)/2}$$ (3)

where $\hat{y_{i}}$ represents the predicted value, *y*_*i*_ represents the true value, and *n* represents the number of samples. The three evaluation indicators measure the closeness of the predicted value to the true value. The closer the value is to 0, the better the prediction effect of the model is.

### 4.2 Prediction effect and robustness analysis of the LSTM model

To further consider the prediction effect of the LSTM model, we use the AttLSTM model [42], SVR model, MLP neural network and random forest model as control models and divide the model prediction period into long-term (94 periods), medium-term (54 periods) and short-term (24 periods) periods to test the robustness of the LSTM model. The control models selected in this work offer different algorithm advantages as shown in Table 5.

**Table 5:** Interpretation of comparison models.

Model	Description
AttLSTM	The attention mechanism layer is added to the LSTM network to make better use of the input data. Comparisons to the ordinary LSTM model are conducted to determine if the weight given to the input can achieve outstanding performance.
SVR	Support vector regression is a regression version of the support vector machine. It is a nonparametric regression technology developed based on the assumption of structural risk minimization, which can achieve good data generalization results. Comparisons to the LSTM model determine if the support vector regression of loss calculation "inclusive" has good performance.
MLP	The multilayer neural network model is a feed-forward neural network model with multilayer perceptrons. The model better fits the relationship between training data by continuously adjusting link weights between neurons. Comparisons to the LSTM model reveal the different effects of the relationships between training data.
Random Forest	The random forest algorithm (regression) is a supervised ensemble algorithm model. The model is composed of many regression trees, and the results are aggregated from the results of many regression trees. Comparisons to the LSTM model reveal the prediction effect of the integrated model.

The prediction results of the average borrowing interest rate time series of the LSTM model and other control models are shown in Table 6. The prediction results of the average borrowing period time series are shown in Table 7.

**Table 6:** Average borrowing interest rate prediction results.

	long-term (94 periods)			medium-term (54 periods)			short-term (24 periods)		
	RMSE	MAPE	SMAPE	RMSE	MAPE	SMAPE	RMSE	MAPE	SMAPE
LSTM	**0.372**	**15.446%**	**14.114%**	**0.411**	**16.943%**	**15.849%**	0.336	18.064%	16.141%
AttLSTM	0.392	16.673%	14.905%	0.438	20.588%	17.882%	**0.323**	**16.719%**	**15.115%**
SVR	1.578	40.271%	28.033%	1.734	50.903%	33.132%	1.585	41.365%	28.204%
MLP	1.753	44.369%	27.385%	2.039	55.144%	33.624%	1.852	44.716%	27.937%
Random Forest	1.934	66.483%	41.230%	2.439	64.689%	45.410%	2.262	63.400%	35.706%

Note: The part in bold is the model’s evaluation indicator results with the best prediction effect.

**Table 7:** Average borrowing period prediction results.

	long-term (94 periods)			medium-term (54 periods)			short-term (24 periods)		
	RMSE	MAPE	SMAPE	RMSE	MAPE	SMAPE	RMSE	MAPE	SMAPE
LSTM	0.280	15.072%	13.129%	**0.295**	17.077%	**14.493%**	**0.211**	**11.874%**	11.535%
AttLSTM	**0.276**	**13.496%**	**12.287%**	0.316	**16.762%**	14.757%	0.215	12.057%	**11.439%**
SVR	0.796	37.433%	27.664%	0.781	29.934%	26.867%	0.759	34.754%	29.962%
MLP	0.864	39.625%	31.651%	0.997	50.198%	36.946%	1.049	35.339%	37.890%
Random Forest	0.877	33.005%	34.944%	0.959	31.544%	35.153%	0.779	34.484%	36.245%

Note: The part in bold is the model’s evaluation indicator results with the best prediction effect.

Table 6 reports the prediction effects of different prediction models for the average borrowing rate for the long, medium, and short term. The average RMSE of the LSTM model for the three prediction periods is 0.373, the average MAPE is 16.82%, and the average SMAPE is 15.37%, which are lower than the average of the corresponding indicators of other control models. Therefore, the LSTM model has better predictive ability than other control models.

Across different prediction periods, evaluation indicators RMSE, MAPE and SMAPE are lower than other control models in the long- and medium-term average borrowing interest rate predictions of the LSTM model. In the short-term average borrowing interest rate prediction, the three evaluation indicators of the AttLSTM model are lower than those of the LSTM model and the other control models. However, the three evaluation indicators of the LSTM model are still significantly lower than those of the SVR, MLP and random forest models, which shows that the LSTM-type models (LSTM and AttLSTM) offer apparent advantages in prediction ability relative to SVR, MLP and random forest models.

Table 7 reports the prediction effects of the prediction models for the average borrowing period for the long, medium, and short term. First, the average RMSE of the LSTM model for the three prediction periods is 0.262, which is lower than that of the other control models. Second, the average MAPE of the LSTM model for the three prediction periods is 14.67%, which is 4.04% higher than the 14.11% value found for the AttLSTM model but lower than the values of the SVR, MLP and random forest models. Finally, the average SMAPE of the LSTM model across the three prediction periods is 13.05%, which is 1.75% higher than the 12.83% value found for the AttLSTM model but still lower than the values of the SVR, MLP and random forest models. Therefore, the LSTM-type models (LSTM and AttLSTM) are better than the SVR, MLP and random forest models in predicting the average loan period.

For different prediction periods, evaluation indicators RMSE, MAPE and SMPE are higher than the AttLSTM model result in terms of the long-term average loan period prediction of the LSTM model but significantly lower than values of the SVR, MLP and random forest models. For medium-term average loan term prediction, the MAPE of the three evaluation indicators of the LSTM model is higher than that of the AttLSTM model but significantly lower than values of the SVR, MLP and random forest models, while the other two indicators (RMSE and SMAPE) are lower than those of the other control models. In the short-term average loan term prediction, the SMAPE of the three evaluation indicators of the LSTM model is higher than that of the AttLSTM model but significantly lower than the values of the SVR, MLP and random forest models, while the other two indicators (RMSE and MAPE) are lower than those of the other control models.

From Tables 6 and 7, the LSTM-type models (LSTM and AttLSTM) have more substantial prediction effects than the SVR, MLP and random forest models. Between the LSTM-type models, the LSTM model performs better than the AttLSTM model in 10 of the 18 prediction results (55.56%), and the AttLSTM model performs better than the LSTM model in 8 of the 18 prediction results (44.44%). In addition, from the specific results of the three evaluation indicators, the sum of the specific numerical results of the 9 prediction results of the average borrowing interest rate (Table 6) of the LSTM model is 2.085, which is 4.01% lower than that of the AttLSTM model. The sum of the specific numerical results of the 9 prediction results of the average loan period (Table 7) of the LSTM model is 1.338, which is 0.07% lower than that of the AttLSTM model. Therefore, the overall prediction effect of the AttLSTM model is weaker than that of the LSTM model.

Given the LSTM models’ (LSTM and AttLSTM) excellent prediction performance, we compare their robustness. Intuitively, Tables 6 and 7 show that the LSTM model presents no apparent fluctuations in the prediction results of the two quantitative indicators of the P2P market across different prediction periods. The data results of the three evaluation indicators are also relatively similar. After specific calculations, the difference between the prediction results of the LSTM model across the two quantitative indicators of different prediction periods is controlled at 30.47%, which is lower than that of the AttLSTM model. Therefore, the LSTM model is also superior to the AttLSTM model in terms of model robustness.

In summary, the LSTM model is superior to other control models in terms of predictive effects and robustness.

## 5 Conclusions and recommendations

We use investor sentiment to predict the average borrowing interest rate indicator and average borrowing period indicator of the P2P market with positive results. From the empirical results of model prediction, the LSTM models (LSTM and AttLSTM) are better than the control SVR, MLP and random forest models at predicting the two quantitative indicators of the P2P market. Between the LSTM models (LSTM and AttLSTM), the comprehensive performance of the LSTM model’s prediction effect and model robustness is better than that of the AttLSTM model, demonstrating the excellent performance of the LSTM model in applying the empirical data used in this article.

In terms of specific indicators, the percentage of positive comments on investor sentiment has a positive relationship to the borrowing interest rate of investors willing to participate in bidding projects on P2P platforms. The percentage of negative comments with investor sentiment has an inverse relationship to the borrowing period of P2P platforms. Therefore, when the positive comments of P2P lending investors increase, investors in the P2P market believe that P2P platforms are less risky. Given the assumptions of rational people in economics, investors will prefer to choose projects with a higher lending interest rate in investment decisions to achieve profit-seeking goals. However, this may also lead to the blind selection of borrowers and cause losses. When the negative comments of P2P lending investors increase, investors in the P2P market believe that the risks of P2P platforms are more remarkable, and they will prefer to choose projects with short borrowing periods to avoid dangers of investment choices.

The development of P2P lending is a microcosm of the new financial format under the blessing of fintech. The difficulties of P2P platform risk screening and investors’ ignorance of the new financial format’s risks make the development of this industry highly uncertain. Based on the above analysis of the quantitative indicators of the P2P market, this article makes the following suggestions for the development of China’s new financial formats.

First, regulators should improve the regulatory system for new financial formats and set industry access standards. As far as China’s P2P industry is concerned, the industry’s average borrowing interest rate and average borrowing maturity indicators are important indicators for distinguishing between problematic platforms and normal platforms [4]. In formulating industry access standards, special attention should be given to better supervising the industry. For example, regulators should set a uniform industry standard range for the product interest rate. It is necessary to ensure industry standards, leave room for innovation, minimize adverse selection and moral hazard, and present inferior products with high interest rates from expelling high-quality products with relatively low interest rates from the market. In addition, the product period setting should also be checked to ensure that it maintains certain industry standards. Regulators need to have new financial formats be explained in the transaction descriptions so that investors can fully understand product transaction risks and precautions. Regulators must also coordinate information symmetry between borrowers and lenders to promote smooth transactions in the market.

Therefore, in accordance with the experiences of the P2P industry, other new financial formats should also focus on grasping the important indicators of their respective product transactions, strengthening the supervision of industry access standards, providing basic guarantees for the development of new financial formats, and preventing the emergence of P2P platform circumstances wherein investors suffer losses due to abnormal indicators.

Second, regulators should make full use of the advantages of big data and dynamically employ the indicator forecasting method. An industry’s quantitative indicator prediction can grasp industry dynamics in time, which should must be monitored by industry big data. Taking this article as an example, we can formulate industry indicator monitoring standards for different periods based on updates of LSTM model forecast data, update standards in development, guide directions of development, make full use of the advantages of financial technology, and dynamically explore development strategies for new financial formats.

Third, regulators should attempt to establish a data linkage mechanism to promptly warn of the risks of new financial formats. In the digital economy era, various kinds of statistics have penetrated every aspect of people’s lives. New financial formats can be used to establish linkage mechanisms with data in other fields to use data models to analyze investor credit conditions under the framework of big data and provide timely warning feedback.
